# Effects of COVID-19 pandemic on pediatric tuberculosis: decrease in notification rates and increase in clinical severity

**DOI:** 10.1007/s00431-023-04981-7

**Published:** 2023-05-09

**Authors:** Andrea Lo Vecchio, Sara Maria Scarano, Chiara Amato, Maria Immacolata Spagnuolo, Eugenia Bruzzese, Alfredo Guarino

**Affiliations:** Department of Translational Medical Sciences - Section of Pediatrics, University of Naples “Federico II”, Via Pansini 5, 80131 Naples, Italy

**Keywords:** Tuberculosis, Children, Infection rate, Disease severity, COVID-19

## Abstract

The outbreak of COVID-19 resulted in a decrease in tuberculosis notification rates globally. We compared tuberculosis incidence rates and disease severity in children seen in our centre prior and during COVID-19 pandemic.We performed a cohort study enrolling children aged under 18 years who received a diagnosis of tuberculosis (January 1st, 2010-December 31st, 2021) at our Pediatric Infectious Diseases Unit. Disease severity was evaluated based on: the classification proposed by Wiseman et al., smear positivity, presence of symptoms at presentation, lung cavitation, extrapulmonary disease, respiratory failure and need for intensive care support. Overall, 168 children (50.6% female, median age 69 months, IQR 95.4) received a diagnosis of tuberculosis, 156 (92.8%) between 2010–2019, before COVID-19 outbreak, and 12 (7.2%) between 2020–2021, during the pandemic. The annual tuberculosis notification rate dropped by 73% in 2021 (0.38/100000, 95%CI 0.1–0.96) compared with 2019 (1.46/100000, 95%CI 0.84–2.37). Compared to the pre-pandemic period, the proportion of children classified as severe was higher in 2020–2021 (5, 41.6% vs 23, 15.7%, p = 0.006) with a higher rate of respiratory failure (2, 16.7%, vs 4, 2.6%, p = 0.01) and an increased need for intensive care support (1, 8.3% vs 1, 0.6%, p = 0.01).

*Conclusion*: During COVID-19 pandemic we observed a reduction in tuberculosis notification rate in pediatric population and a significant increase in disease severity. This scenario may be the consequence of a delay in diagnosis and an underreporting of cases, rather than the effect of a reduced transmission of tuberculosis. Children reached health-care services only in the need of urgent medical attention.

**Table:** 

**What is Known:** *• COVID-19 pandemic had a huge impact on national health care systems, resulting in a reduction of access to medical care.*	
**What is New:** *• In Campania Region, Italy, a low tuberculosis incidence country, we witnessed a 75% reduction in tuberculosis notification rate during pandemic. In parallel we demonstrated a significant increase in disease severity, suggesting that the reduction in notification rate may be attributed to an underreporting of cases and consequential diagnostic delay, rather than a reduced transmission of infection.*

**What is Known:**

*• COVID-19 pandemic had a huge impact on national health care systems, resulting in a reduction of access to medical care.*

**What is New:**

*• In Campania Region, Italy, a low tuberculosis incidence country, we witnessed a 75% reduction in tuberculosis notification rate during pandemic. In parallel we demonstrated a significant increase in disease severity, suggesting that the reduction in notification rate may be attributed to an underreporting of cases and consequential diagnostic delay, rather than a reduced transmission of infection.*

## Introduction

The outbreak of Coronavirus Disease (COVID-19) resulted in a decrease in tuberculosis (TB) notification rates globally.

As a reference center for pediatric TB in a Southern Italy populous region, we observed a drop in TB diagnoses that was countertrend compared to the last ten years of observation [1]. This reduction in TB notification might be the consequence of an underreporting of cases and a delay in diagnosis, that could have affected disease severity at presentation and clinical outcome.

To test this hypothesis, we hereby compare TB incidence rates, disease severity and outcome in children seen prior and during COVID-19 pandemic.

## Methods

We performed a single center cohort study enrolling all children (< 18 years) who received a microbiologically confirmed or unconfirmed diagnosis of TB between January 1^st^, 2010 and December 31^st^, 2021 at our Pediatric Infectious Diseases Unit. Data was collected by using a prospective database as previously reported [1].

TB disease was defined according to established consensus criteria [2]: i) confirmed TB (microbiological confirmation by culture or polymerase chain reaction), or ii) unconfirmed TB: presence of at least two of the following: a) symptoms/signs suggestive of TB, b) chest radiograph consistent with TB, c) known TB exposure or immunologic evidence of TB infection, d) positive response to TB treatment. Children exposed to TB or diagnosed with latent TB infection were excluded. Patients were classified as affected by pulmonary or extrapulmonary TB according to the European Centre for Disease Prevention and Control criteria. Those with multiple disease localizations were classified considering the most severe clinical manifestation.

Disease severity was evaluated based on the following: the classification proposed by Wiseman et al. according to localization, extent and complexity of TB disease [3], smear positivity, presence of symptoms at presentation, lung cavitation, extrapulmonary disease, respiratory failure and need for intensive care support. The definition of poor clinical outcome was based on the need of a prolonged TB treatment (at least one month beyond usual indications), the presence of sequelae, relapse and death.

As the PIDU is the unique regional referral center, TB rates were calculated dividing the number of new cases diagnosed at the PIDU with the total number of residents below 18 years of age in the Campania Region, according to the Italian Institute of Statistics.

Results were compared by using t-test or MannWhitney nonparametric test, as appropriate. Categorical variables were summarized and reported as frequencies and percentages and compared through Fisher exact test or χ2 test, as appropriate.

## Results

Overall 168 children (50.6% female, median age 69 months, IQR 95.4) received a diagnosis of TB during the study period, 156 (92.8%) between 2010–2019, before COVID-19 outbreak, and 12 (7.2%) between 2020–2021, during the pandemic (Table 1). The majority of children (128, 76.2%) had pulmonary TB, with no significant difference between the two periods (58.3% vs 77.5%, p = 0.13), and 38 (22.6%) had microbiological confirmation.

**Table 1:** Incidence, clinical features and outcomes of children with TB (n = 168) in pandemic and pre-pandemic period

	**Pandemic (2020–2021)** **TB cohort**	**Pre-pandemic (2010–2019) TB cohort**	***p***	**2-year** **pre-pandemic** **(2018–2019) TB cohort**	***p***
TB incident cases	12	156	-	38	-
TB rate/100.000 inhabitants (95%CI)	0.56 (0.01–1.25)	1.17 (0.24–2.10)	-	1.66 (0.49–2.83)	-
Mean number of cases/year (SD)	6.0 (2.8)	15.6 (4.0)	0.058	19.0 (4.3)	0.042
Female gender (%)	7 (58.3)	78 (50.0)	0.577	22 (57.8)	0.978
Foreign origin (%)	9 (75.0)	77 (49.3)	0.086	20 (52.6)	0.171
**Reason for TB testing**					
Contact tracing	5 (41.7)	99 (63.4)	0.13	29 (76.3)	0.02
Symptoms	7 (58.3)	53 (34.0)	0.08	7 (18.4)	0.007
Migrant screening/adopted children	0 (0.0)	4 (2.6)	1.00	2 (5.3)	1.00
**Case definition**					
Microbiologically confirmed (%)	4 (33.3)	34 (21.7)	0.357	13 (34.2)	0.358
- Ziehl–Neelsen-positive (%)	1 (8.3)	13 (8.3)	1.00	6 (15.8)	0.516
- Gene-X-pert-positive (%)	4 (33.3)	33 (21.1)	0.005	12 (31.5)	0.909
- Culture positive (%)	2 (16.7)	23 (14.7)	0.856	8 (21.0)	0.740
**Drug resistance**					
Sensitive to first-line treatment (%)	12 (100.0)	146 (93.5)	1.00	36 (94.7)	1.00
Isoniazid/Rifampin resistant (%)	0 (0.0)	5 (3.2)	1.00	1 (2.6)	1.00
Multi drug resistant (%)	0 (0.0)	5 (3.2)	1.00	1 (2.6)	1.00
**Primary localization**					
Pulmonary (%)	7 (58.3)	121 (77.5)	0.131	32 (84.2)	0.059
Pleuro-pulmonary (%)	3 (25.0)	10 (6.4)	0.020	2 (5.3)	0.046
Bronchial/laryngeal (%)	0 (0.0)	2 (1.3)	1.00	0 (0.0)	1.00
Lymph Nodes (%)	1 (8.3)	10 (6.4)	0.795	3 (7.9)	0.160
Osteo-articular (%)	0 (0.0)	4 (2.6)	1.00	0 (0.0)	1.00
Intestinal (%)	0 (0.0)	3 (1.9)	1.00	0 (0.0)	1.00
Miliary/meningeal (%)	0 (0.0)	6 (3.8)	1.00	1 (2.6)	1.00
Other (%)	1 (8.3)	0 (0.0)	1.00	0 (0.0)	1.00
**Disease severity**					
Severe cases according to Wiseman’s classification (%)	5 (41.6)	23 (14.7)	0.006	4 (10.5)	0.014
Symptoms at presentation (%)	7 (58.3)	102 (65.4)	0.621	24 (63.1)	0.764
Smear-positive (%)	1 (8.3)	13 (8.3)	1.00	6 (15.8)	0.516
Lung cavitation (%)	2 (16.7)	8 (5.1)	0.104	3 (7.9)	0.377
Respiratory failure (%)	2 (16.7)	4 (2.6)	0.011	1 (2.6)	0.074
Extra-pulmonary disease (%)	5 (41.6)	35 (22.4)	0.131	6 (15.8)	0.059
Need for intensive care support (%)	1 (8.3)	1 (0.6)	0.017	0 (0.0)	0.072
**Clinical outcome**					
Healing	5 (41.6)	89 (57.0)	0.300	17 (44.7)	0.851
Poor outcome	7 (58.3)	48 (30.7)	0.049	16 (42.1)	0.325
- Need for prolonged treatment/drug shift (%)	6 (50.0)	34 (21.8)	0.027	10 (26.3)	0.125
- Sequelae (%)	1 (8.3)	6 (3.8)	0.453	3 (7.9)	0.160
- Relapse (%)	0 (0.0)	6 (3.8)	1.00	3 (7.9)	1.00
- Death (%)	0 (0.0)	2 (1.3)	1.00	0 (0.0)	1.00
Lost to follow-up	0 (0.0)	19 (12.1)	0.364	5 (13.1)	0.319

The mean number of incident cases per year decreased from 15.6 ± 4.0 in the pre-pandemic period to 6 ± 2.8 in 2020–2021 (p = 0.058), and the annual TB notification rate dropped by 73% in 2021 (0.38/100000, 95%CI 0.1–0.96) compared with 2019 (1.46/100000, 95%CI 0.84–2.37).

During the pandemic a higher proportion of children came to testing for TB and received diagnosis because of the onset of symptoms (7, 58.3%) compared to the two years pre-pandemic 2018–2019 (7, 18.4%, p = 0.007), without reaching statistical significance when compared to 2010–2019.

The proportion of children classified as severe was higher in 2020–2021 (5, 41.6%) compared with 2010–2019 (23, 14.7%, p = 0.006) (Table 1).

Compared to the pre-pandemic period, children diagnosed with TB during COVID-19 pandemic showed a higher rate of respiratory failure (2, 16.7%, vs 4, 2.6%, p = 0.01), an increased need for intensive care support (1, 8.3% vs 1, 0.6%, p = 0.01) and a higher proportion of poor clinical outcome (7, 58.3% vs 48, 30.7%, p = 0.04).

The two-years pre-pandemic (2018–2019) comparative analysis confirmed a statistically significant increase in the proportion of severe TB cases during the pandemic (2020–2021) (Table 1), although the rates of respiratory failure, intensive care admission and poor outcome did not reach statistical significance.

Children receiving a TB diagnosis during COVID-19 pandemic had a significantly higher risk of presenting with severe disease, compared with those diagnosed in 2010–2019 (OR 3.93, 95%CI 1.15 to 13.4, p = 0.028) and also with those diagnosed in the last two years before the pandemic (OR 6.07, 95%CI 1.29 to 28.5, p = 0.022).

## Discussion

During COVID-19 pandemic a marked decrease in TB notification rate has been reported at European [4] and global level [5]. However, the effects that pandemic restrictions had on children living in European countries are unknown.

The present study revealed a 70-percent drop in TB incident cases among children living in a low-incidence European Country, and demonstrated a parallel rise in the severity of the disease during the first two years of COVID-19 pandemic.

The decrease in the number of new TB diagnoses could have been either the effect of a reduced transmission of TB, as an effect of Severe Acute Respiratory Syndrome CoronaVirus 2 (SARS-CoV-2) containment measures, or the consequence of a delay in diagnosis and an underreporting of cases.

In Italy the overall TB notification rate decreased by about one third in 2020 [4], which could reflect COVID-19 containment measures, such as social distancing and face masks, leading to lower TB transmission in the community. Before the pandemic, mask use among TB patients was poor, due to the fear of stigma, and only 2% of drug-resistant TB patients reported wearing surgical masks in public areas [6].

The effect of reduced transmission in the community is likely to be observed earlier in children, who usually progress faster to TB disease than adults following primary *M. tuberculosis* infection. On the other hand, staying at home could have accelerated transmission within households, an important aspect in children for whom the index case is typically an adult household member.

During the pandemic, children had reduced access to medical care due to the parents’ fear of being infected with SARS-CoV-2 in hospital settings, to the barriers towards direct visits by primary care physicians, and to the reallocation of health-care personnel toward the COVID-19 units. Migliori et al. reported a reduction of TB services based on data collected from 33 centers located in 16 countries worldwide [7]. McQuaid CF et al*.* suggested that, although social distancing measures can be beneficial in settings where health services are less affected, resulting in a lower number of TB cases, this beneficial effect is more likely to be outweighed by health service disruption, especially in low- and middle-income settings [8].

In Campania region, a populous region in Southern Italy, we previously observed a significant and progressive increase in TB notification rates between 2009 and 2018. This phenomenon was directly associated with migration [1]. Notably, the drop in TB diagnosis observed during the pandemic appeared unrelated to migration flows in the region, where the rate of migrants concomitantly increased, reaching 4% of the resident pediatric population [9]. During the pandemic, the risk of TB exposure in migrants was limited by restrictions to visit friends and relatives in high TB incidence countries, however we actually observed a high proportion of children of foreign origin among TB diagnoses. While this could be related to a lack of Italian TB cases, the magnified difficulties in accessing medical care for migrant populations must be taken into account and the number of TB cases of foreign origin might even be underestimated.

In addition, for children seeking care for respiratory symptoms and fever, the priority was to exclude SARS-CoV-2 infection, rather than performing detailed diagnostics for other infections, including TB.

The World Health Organization estimated that the decrease in TB case detection could result in half a million excess TB deaths in the next few years, depending on the degree and duration of disruption in disease detection [10].

During the pandemic, children often reached health services only when they were severely ill and required urgent medical attention, and the few who received a new diagnosis of TB showed compromised clinical conditions and a largely disseminated TB infection. According to our data, receiving a diagnosis during COVID-19 pandemic exposed to a 3- to sixfold increased chance of severe TB disease.

Of note, none of the new patients receiving a diagnosis of TB resulted positive to SARS-CoV-2 tests at admission, and we did not observe any severe SARS-CoV-2 infection requiring hospital care in children in follow-up for TB disease.

No major differences in drug resistance pattern, access to specific treatment or adherence to follow-up were observed in the cohorts.

Notably, some centers reported increased follow-up losses during pandemic [11]. We actually observed that the main barrier to hospital access for new TB patients was related to caregivers’ hesitancy to reach the hospital during pandemic, rather than being related to a shortage of health-care personnel. In our institution, the same specialists who took care of COVID-19 patients were involved in the management of children with other infections (including TB patients managed in a different hospital area). Hence, we have been able to keep follow-up service active during pandemic, rescheduling any missed appointments as soon as possible.

This supports our hypothesis that the delay in TB diagnosis and the underreporting are the major determinants of the increased disease severity and more severe clinical outcome observed in our population.

Although in 2021 most restrictive measures were dismantled and TB services were completely restored in the region, the TB notification rate was even lower than that in 2020. This indicates that the restoration of standard services might be not sufficient, and it raises a worrisome question: “how long would it take to track and treat all undetected cases of TB that we missed during the pandemic?”.

Supplementary measures are urgently required to attenuate the impact of COVID-19 pandemic in order to catch-up with the missed diagnoses.

## Data Availability

The corresponding author is responsible for the collection of data, their anonymization, management and presentation. The entire database of the present study is available on appropriate and motivated request.

## Abbreviations

COVID-19: 2019-CoronaVirus Disease
SARS‑CoV‑2: Severe Acute Respiratory Syndrome CoronaVirus 2
TB: Tuberculosis
